# Multiple congenital malformations in a burrowing owl (*Athene cunicularia*) from Southern Brazil

**DOI:** 10.1007/s11259-026-11531-7

**Published:** 2026-09-19

**Authors:** Giovanna Pugioli Comine, Eduarda Aléxia Nunes Louzada Dias Cavalcanti, Rafael Kretschmer, Matheus Giannechini Medeiros, Raqueli Teresinha França

**Affiliations:** 1 Wildlife Rehabilitation Centre (NURFS-CETAS), Federal University of Pelotas (UFPel), Campus Capão do Leão, Pelotas, Rio Grande do Sul CEP 96010-900 Brazil; 2 Cytogenetics and Evolution Laboratory, Department of Genetics, Biosciences Institute, Federal University of Rio Grande do Sul (UFRGS), Porto Alegre, Rio Grande do Sul Brazil

**Keywords:** Developmental anomalies, Limb deformities, Raptors, Strigiformes, Teratogenesis, Wildlife health

## Abstract

This study reports a case of multiple congenital malformations in a burrowing owl (*Athene cunicularia*) rescued in southern Brazil. Clinical assessment revealed partial agenesis of the forelimbs, bilateral asymmetric syndactyly, and selective anonychia of the second digit in both pelvic limbs. Despite severe locomotor limitations and the inability to fly, the individual maintained a satisfactory body condition score, demonstrating the species’ adaptive and behavioural capacity. The integrity of the epithelial tissues and the symmetry of the digital alterations support a teratogenic etiology, distinguishing the condition from traumatic lesions. The absence of macroscopic chromosomal abnormalities suggests that these anomalies may be associated with environmental factors or point mutations. This report highlights the importance of wildlife rehabilitation and triage centers in detecting sentinel alterations, serving as an alert for wildlife conservation regarding potential environmental contaminants that may impair embryonic development in anthropogenic landscapes.

## Background

The burrowing owl (*Athene cunicularia*) is a strigiform bird belonging to the family Strigidae. It exhibits predominantly terrestrial habits (Ilyinsky 2008), characterized by cursorial behavior and burrowing ability (Zarn 1974; WikiAves 2026). Its wide geographical distribution extends from southern Canada to the southernmost regions of South America, and the species is resident throughout Brazil (BirdLife International 2016; WikiAves 2026). Recognized for its ecological plasticity, this species occupies a wide range of open habitats, including grasslands, savannas, dunes, and urbanized areas (Zilio 2005; Andery et al. 2013; BirdLife International 2016; Silva and Donatelli 2024; Jones et al. 2025). Consequently, it has been the subject of numerous ecological and conservation studies across the Americas (Silva and Donatelli 2024). Despite extensive ecological knowledge, gaps remain regarding its anatomophysiology (Kilgore et al. 2008) and the occurrence of congenital or structural malformations, which remain poorly explored in the literature (Gervais et al. 2007; Pourlis 2011; Andery et al. 2013).

In different regions of the Northern Hemisphere, acquired conditions, such as traumatic injuries and infectious processes affecting the pelvic limbs and digits, have been documented in burrowing owls (Gervais et al. 2007; Oliveira et al. 2025), alongside parasitic infections in urban populations (Jones et al. 2025). In Brazil, abnormalities in birds of prey admitted to wildlife rehabilitation centers are occasionally reported (Andery et al. 2013; Lima et al. 2026), with traumatic injuries being the most frequent. Conversely, congenital malformations, particularly those affecting the thoracic limbs, remain extremely rare and poorly documented in wild populations. These anomalies may include the total or partial absence of limbs, duplication of structures (polymelia and diplopodia), hypoplasia, skeletal deformities, and digital fusions such as syndactyly. Although more commonly reported in domestic species, their occurrence in wild birds is likely underreported because affected individuals have a low probability of survival in nature. Investigating congenital abnormalities is therefore highly relevant, as they directly impact individual fitness, survival, and population dynamics (Pourlis 2011; Orosz et al. 2023). This study describes a case of multiple congenital malformations in a subadult specimen of *Athene cunicularia* from Southern Brazil, integrating clinical, radiographic, and cytogenetic findings.

## Case presentation

In October 2024, a burrowing owl (*Athene cunicularia*), presenting plumage characteristics consistent with a juvenile individual (Poulin et al. 2020) and weighing 132 g, was found in the municipality of Rio Grande, Southern Brazil, and subsequently admitted to the Wildlife Rehabilitation Centre and Wild Animal Triage Centre of the Federal University of Pelotas (NURFS-CETAS/UFPel). At admission, physical examination revealed a cloacal temperature of 39 °C, normal mucous membranes, estimated dehydration of 8%, blood glucose of 280 mg/dL, and a body condition score of 3 (on a scale of 1–5).

**Fig. 1:**
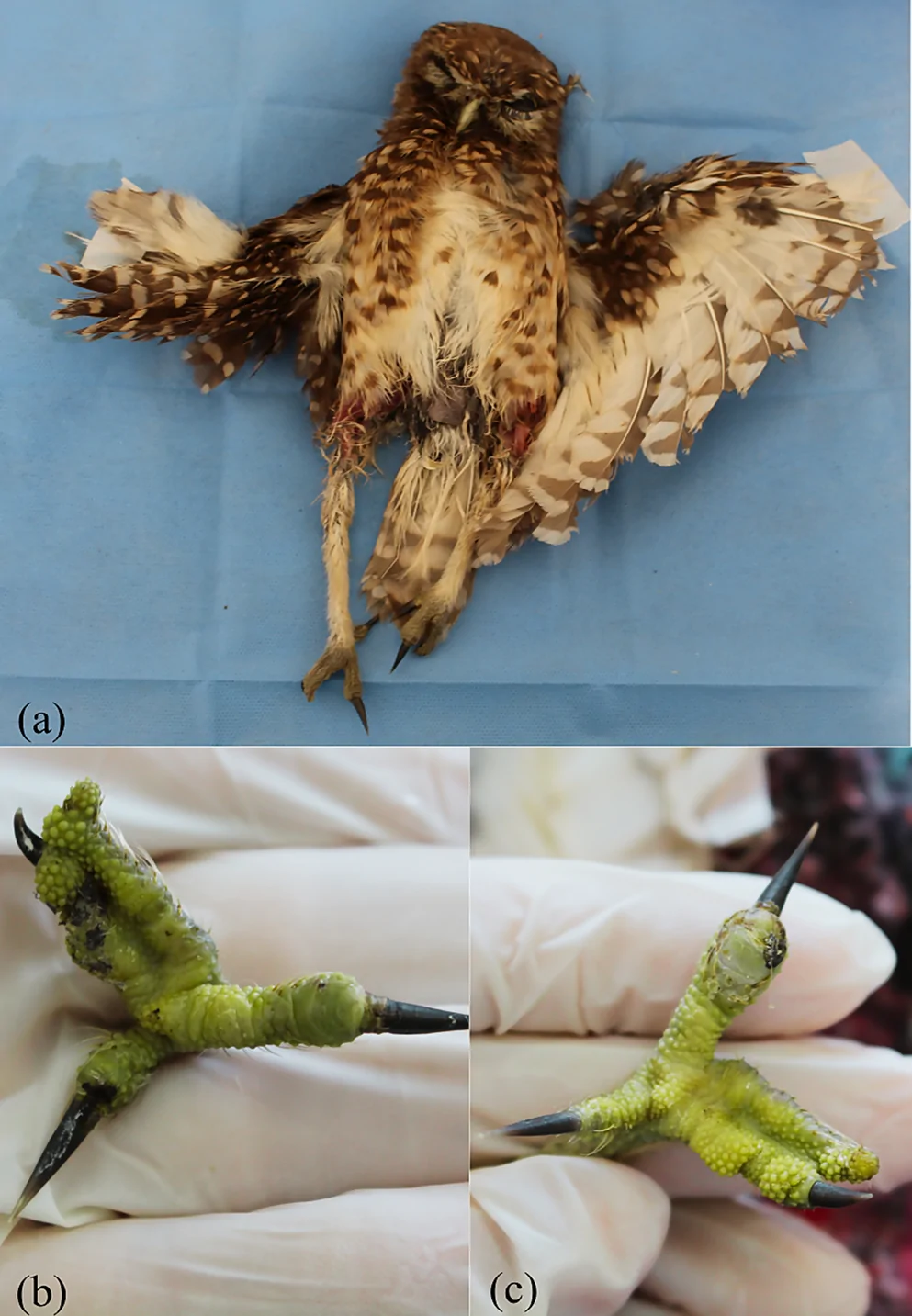
Post-mortem photographs of morphological alterations in *Athene cunicularia*. (**a**) Asymmetry between the wings, with marked reduction of the right thoracic limb compared with the contralateral limb. (**b**) Plantar view of the right pelvic limb showing syndactyly between digits I and II, associated with the absence of the claw on digit II. (**c**) Plantar view of the left pelvic limb showing syndactyly between digits II and III, also with the absence of the claw on digit II. Continuity of the epithelium in the fusion areas and partial preservation of digital structures can be observed

Macroscopic anatomical abnormalities were observed in the right wing, characterized by a shortening of the radius and ulna, complete absence of the metacarpus, and disorganized remiges. In contrast, the left wing appeared externally preserved upon inspection. Additionally, bilateral asymmetric anomalies were observed in the pelvic limbs. In the right pelvic limb, syndactyly between digits I and II was identified, accompanied by anonychia (absence of claw) of digit II. In the left pelvic limb, syndactyly occurred between digits II and III, also with anonychia affecting digit II. In both limbs, the areas of fusion involved soft tissues with no visible interdigital segmentation. The distal portions of the affected digits exhibited continuous and intact epithelial tissue, with no evidence of inflammation or traumatic avulsion scars (Fig. 1).

**Fig. 2:**
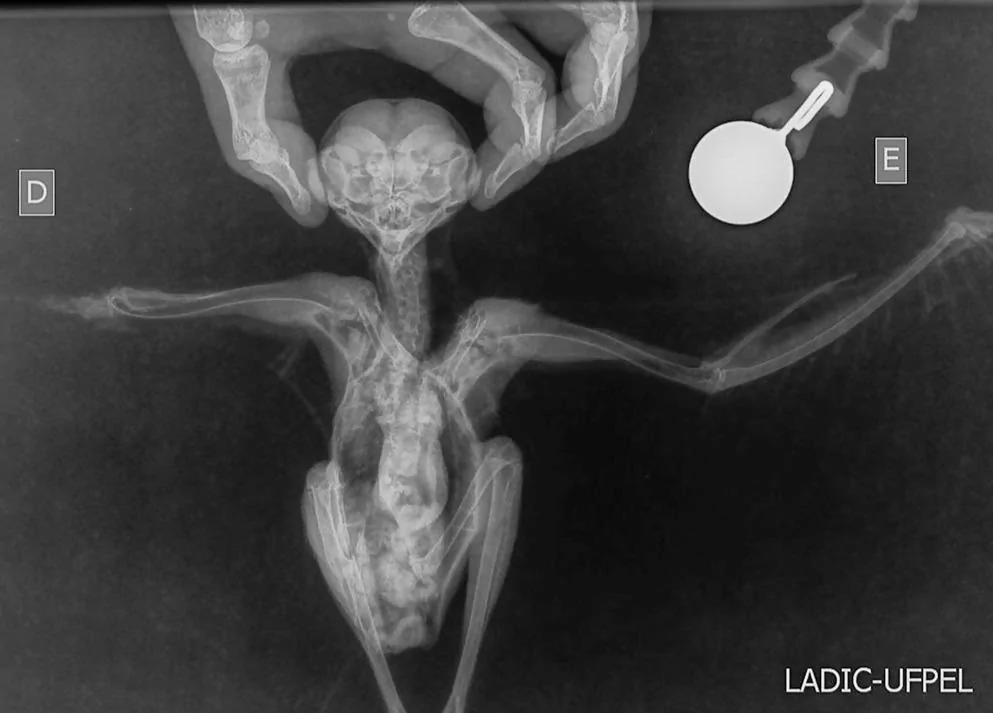
Ventrodorsal whole-body radiographic projection of *Athene cunicularia*. Image obtained at the Diagnostic Imaging and Cardiology Laboratory (LADIC/UFPel). In the right thoracic limb (R), complete agenesis of the radius, ulna, and distal structures is observed. In the left thoracic limb (L), partial absence and thinning of the distal radius are evident. Bilateral absence of all skeletal structures distal to the forearm (carpus, metacarpus, and phalanges) is noted. The absence of a bone callus supports the exclusion of a previous traumatic etiology

Given the phenotypic and skeletal alterations identified during the initial physical examination, a radiographic assessment was performed to further characterize the extent of these anomalies and investigate the presence of findings suggestive of traumatic processes. Mediolateral projections of the radius and ulna were obtained bilaterally using a fixed X-ray unit (Sawae, Altus ST model) at the Diagnostic Imaging and Cardiology Laboratory (LADIC/UFPel) (Fig. 2).

While macroscopic evaluation suggested a shortening of the right forelimb elements, the radiographic examination revealed more profound, definitive skeletal alterations. The imaging confirmed the complete absence of the radius and ulna in the right thoracic limb, as well as partial absence and thinning of the distal portion of the radius in the left thoracic limb. Additionally, there was a bilateral absence of all skeletal structures distal to the radius and ulna, including the carpus, metacarpus, and phalanges (Fig. 2). The absence of a bone callus or periosteal reaction supported the exclusion of a previous traumatic etiology.

To evaluate potential genetic origins, short-term cultures of bone marrow cells were established to obtain metaphase chromosomes, following the protocol described by Cioffi et al. (2026). Bone marrow samples were suspended in 10 ml of RPMI medium containing 0.1 ml of 0.05% colchicine and incubated at 37 °C for 1 h. Following incubation, the suspension was centrifuged at 800 rpm for 8 min, and the supernatant was removed. The cell pellet was resuspended in 10 ml of hypotonic KCl solution (0.075 M) and incubated at 37 °C for 30 min. The suspension was centrifuged again under the same conditions, and the supernatant was discarded. The pellet was fixed in a methanol: acetic acid solution (3:1) and centrifuged at the same speed and duration. The fixation step was repeated three times to ensure proper cell preservation. The cell suspension was then dropped onto clean slides, air-dried, and stained with 5% Giemsa in 0.07 M phosphate buffer (pH 6.8) for 5 min.

**Fig. 3:**
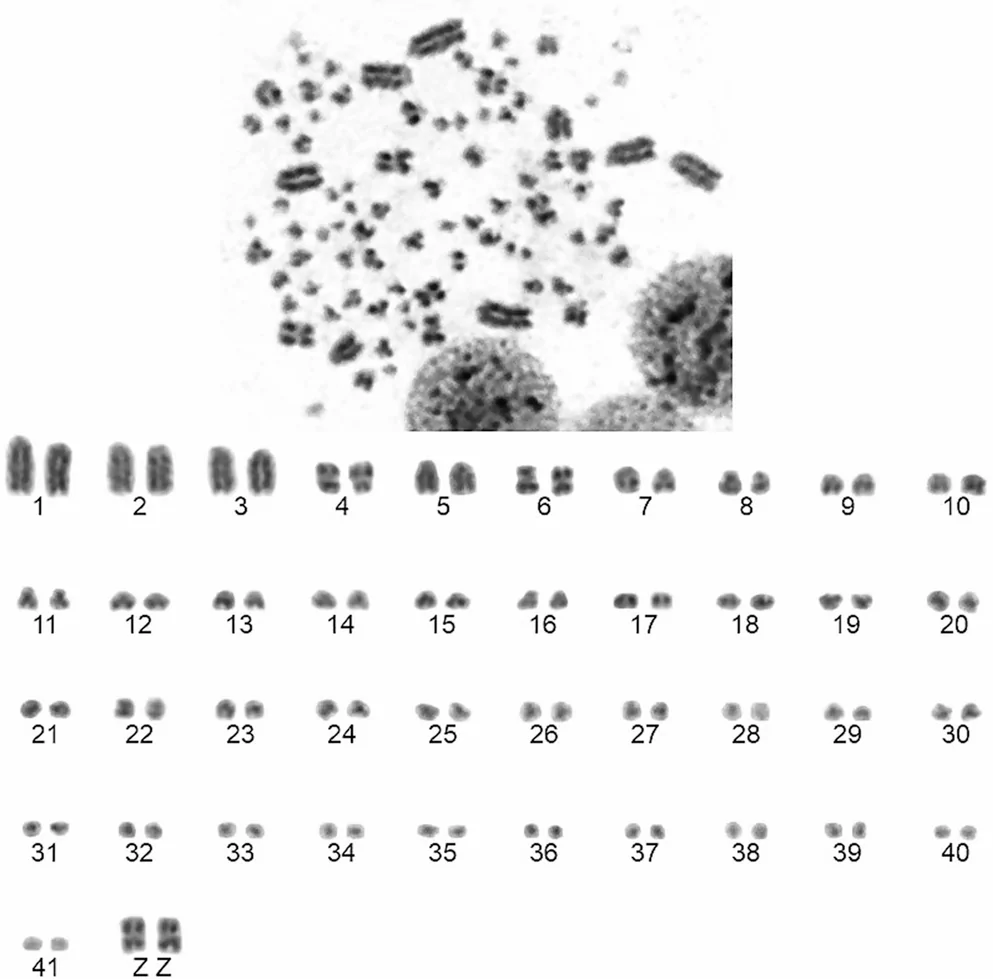
Complete karyotype of *Athene cunicularia*, showing the full chromosomal complement (2*n* = 84) and the ZZ sex chromosome constitution, consistent with a male individual

The diploid number and chromosomal morphology were determined by analyzing 50 metaphase spreads using a ZEISS Axiophot Epifluorescence Microscope (Zeiss, Oberkochen, Germany) equipped with ZEN 2 (Blue edition) software. To ensure accuracy, at least 30 high-quality metaphase spreads were examined to confirm the diploid number and chromosomal morphology. The analyzed individual showed a stable karyotype consisting of 84 chromosomes (Fig. 3), with no chromosomal abnormalities observed in comparison with previously described normal karyotypes for the species (Rebholz et al. 1993; Nogueira and Alves 2011).

Given the severity of the anatomical malformations, which resulted in a permanent inability to fly or hunt, the prognosis for release into the wild was considered poor. Consequently, in accordance with veterinary ethical guidelines and animal welfare considerations, humane euthanasia was performed using standard pharmaceutical protocols. A complete necropsy was performed following euthanasia. Macroscopic examination of the heart, lungs, kidneys, and brain revealed no gross abnormalities. The alterations were restricted to the appendicular skeleton and digits, as previously described.

## Discussion and conclusions

The phenotypic alterations observed in *Athene cunicularia* in the present study exhibit a complex and likely multifactorial origin. Considering that cytogenetic analysis did not reveal numerical or macroscopic structural chromosomal abnormalities, the etiology of these malformations should be discussed in the context of gene-level alterations, submicroscopic damage, or environmentally mediated epigenetic factors (Hoffman 1990; Pourlis 2011; Lamonica et al. 2024). The absence of detectable karyotypic abnormalities does not exclude a genetic basis, as point mutations or submicroscopic alterations undetectable by conventional cytogenetic analysis may be involved (King et al. 2008; Wu et al. 2021). Congenital malformations in birds may arise from interactions between genetic and environmental factors, including exposure to chemical contaminants, nutritional deficiencies, and disturbances during embryonic development (Hoffman 1990; Koivula and Eeva 2010).

Alterations in limb development, including bone shortening, absence of structures, and interdigital fusion, may be linked to disruptions in the expression of regulatory genes involved in embryonic patterning, particularly those controlling the proximodistal and anteroposterior axes (Seki et al. 2012; Umair et al. 2018). Syndactyly, as observed in the present case, may result from a failure of interdigital apoptosis during embryogenesis (Umair et al. 2018). Additionally, environmental factors may act as epigenetic modulators, altering gene expression without modifying the DNA sequence and contributing to abnormal phenotypic outcomes (Lamonica et al. 2024). In free-ranging birds, exposure to environmental pollutants and adverse conditions may play a relevant role in the occurrence of morphological anomalies, although establishing a direct causal relationship remains challenging (Pourlis 2011).

Although no chromosomal alterations were detected by conventional karyotype analysis, this approach cannot exclude other forms of genomic damage that may not be detectable by standard cytogenetic methods. In birds, the micronucleus (MN) assay and the assessment of other erythrocyte nuclear abnormalities have been successfully applied as biomarkers of chromosomal and genomic damage associated with environmental contamination (Baesse et al. 2015; Souto et al. 2025; Cortés-Gutiérrez et al. 2026). Studies conducted in wild birds inhabiting agricultural environments have reported increased frequencies of MN and other nuclear abnormalities in individuals exposed to potentially contaminated habitats, supporting the use of this biomarker as complementary indicators of environmental genotoxicity (Baesse et al. 2015; Souto et al. 2025). Thus, although the normal karyotype observed in the present case provides no evidence of chromosomal abnormalities, the possibility of other forms of genetic damage cannot be completely excluded. Complementary analyses, such as the micronucleus assay or other molecular and cytogenetic biomarkers, could provide additional information regarding potential genotoxic effects and their possible contribution to the phenotypic abnormalities observed in the studied burrowing owl. Experimental evidence has also demonstrated that pesticide exposure can induce embryotoxic and teratogenic effects during avian development (Uggini et al. 2012), while genotoxic and mutagenic effects of teratogenic agents have been documented during mammalian embryonic development (Rencüzoğulları and Aydın 2018). Environmental contaminants, including pesticides, may represent potential sources of genotoxic stress and should therefore be considered as possible contributing factors rather than established causes of the observed abnormalities.

This scenario is particularly relevant considering the site where the specimen was found. Rio Grande, the municipality of origin of the present case, is a coastal city situated in the estuarine area of the Patos Lagoon, historically shaped by intense port, industrial, and urban development (Wallner-Kersanach et al. 2016). Long-term environmental assessments of this estuarine region have documented persistent contamination by trace metals across multiple compartments (water, sediment, soil, and atmosphere), associated with port, naval, industrial, and urban activities developed over the past decades (Wallner-Kersanach et al. 2016). Elevated concentrations of copper, lead, and zinc have been specifically reported in the port waters of the Rio Grande Channel, near ammonia, petroleum, fertilizer, and fish-processing terminals (Barbosa et al. 2012), while a study of the city’s urban landfills, formed through historical land reclamation of the estuary since the 18th century, revealed that older deposits (18th–19th century) presented elevated lead and mercury, whereas 20th-century deposits showed higher concentrations of cadmium, copper, chromium, and zinc (Conceição 2005). Beyond point-source industrial and urban inputs, agricultural runoff draining into the broader Patos Lagoon basin, which encompasses both Rio Grande and Pelotas, has also been identified as a contributing factor to the overall pollutant load reaching this aquatic system (Decker et al. 2018). This combination of anthropogenic contamination sources is further reflected in local biota: heavy metal analyses of shrimp (*Farfantepenaeus paulensis*) harvested from the Patos Lagoon near Rio Grande have detected lead, copper, and zinc, with lead concentrations in the exoskeleton exceeding the maximum limit established by Brazilian legislation in some samples (Pinto et al. 2013), illustrating an active pathway for trophic bioaccumulation of metals in the local food web. Taken together, this body of evidence supports the biological plausibility of environmental exposure to heavy metals and other teratogenic contaminants as a contributing factor to the congenital malformations observed in the present case.

Exposure of eggs to chemical contaminants, such as pesticides and heavy metals commonly present in agricultural, coastal, and peri-urban environments inhabited by burrowing owls, may interfere with embryonic development through mechanisms of embryotoxicity, endocrine disruption, and oxidative stress (Hoffman 1990; Koivula and Eeva 2010). These contaminants can increase the production of reactive oxygen species (ROS), leading to oxidative damage and disruption of normal developmental processes (Koivula and Eeva 2010). Given the opportunistic and generalist feeding habits of *A. cunicularia*, which include the frequent consumption of insects and small vertebrates in agroecosystems and peri-urban areas (Zarn 1974; Poulin 2003; Zilio 2005; Silva and Donatelli 2024; Moniwa and Donatelli 2025), the ingestion of contaminated prey may represent an important route of bioaccumulation. This process can result in the transfer of contaminants to the eggs, contributing to developmental defects in the offspring (Gervais et al. 2007; Lamonica et al. 2024; Lemos et al. 2024). Consequently, environmental contaminants should be considered potential contributing factors rather than established direct causes of the observed abnormalities.

The radiographic alterations observed, including unilateral agenesis of the radius and ulna and bilateral absence of distal limb structures (Fig. 2), are consistent with severe developmental disturbances of the limb bud. Similar malformations have been reported in other avian taxa, where failures in early limb development result in longitudinal reductions of appendicular structures (Pourlis 2011; Garcês et al. 2026). Such conditions are rarely documented in free-ranging animals due to low detection rates and high mortality of individuals with severe locomotor impairments (Pollis 2016; Sousa 2023). Comparable anomalies have been described in other bird species, including bilateral shortening of the radius and ulna in griffon vultures (*Gyps fulvus*) and pelvic limb malformations in barn owls (*Tyto alba*), characterized by phalangeal aplasia and micromelia without evidence of trauma (Garcês et al. 2026). The complete absence of the long bones of the thoracic limb, as observed in this case, represents a severe stage of disruption in limb bud differentiation, typically associated with early insults to the zone of polarizing activity (ZPA) (Zeller et al. 2009; Pourlis 2011).

The symmetry of distal alterations and the presence of anomalies affecting multiple appendicular segments support a congenital origin. A traumatic etiology was excluded based on the absence of a bone callus, periosteal reaction, or angular deformities typically associated with healed fractures in raptors (Orosz et al. 2023). In addition, necropsy findings revealed no alterations in internal organs or other visceral systems, with lesions restricted to the appendicular skeleton and digits. This localized distribution pattern is consistent with a developmental insult confined to limb bud differentiation during early embryogenesis, as teratogenic exposures during early limb formation have been experimentally shown to produce localized, stage-dependent limb reductions and digital defects in avian embryos (Therapontos et al. 2009; Pourlis 2011), without necessarily compromising internal organogenesis (Hoffman 1990). The asymmetric pattern of syndactyly, involving digits I–II on the right and II–III on the left, combined with selective bilateral anonychia of digit II, suggests a complex disruption in digital patterning during embryogenesis. Pourlis (2011) suggested that malformations showing variation in localization between limbs indicate heterogeneous effects on the morphogenetic field during mesenchymal differentiation. This process is regulated by molecular signaling gradients and interactions between embryonic organizing centers, which are essential for proper digit formation (Zeller et al. 2009).

The preservation of claws on the hallux (digit I), in contrast to their consistent absence on digit II, suggests a disruption in apical ectodermal ridge (AER) signaling at specific stages of development. The AER plays a critical role in maintaining mesenchymal proliferation and proximodistal limb growth (Zeller et al. 2009; Gilbert 2013). This presentation differs markedly from acquired traumatic or infectious lesions reported in urban raptor populations (Andery et al. 2013), as the symmetry of digit II anonychia and epithelial integrity support a developmental error rather than postnatal environmental injury.

Despite the severity of the malformations, the individual maintained a satisfactory body condition score. This finding is clinically relevant, as it suggests that the bird retained the ability to capture prey despite being unable to fly, likely due to the species’ flexible feeding ecology. Additionally, social interactions cannot be excluded, as *A. cunicularia* exhibits cooperative social behavior, including pair formation or small family groups, particularly during the breeding season (Zarn 1974; Alberta Environment and Sustainable Resource Development 2012). Juveniles may remain partially dependent on parental care for several weeks after leaving the burrow, receiving food during the transition to independence (Millsap and Bear 2000; Zarn 1974). Thus, as the analyzed individual was a juvenile, it may have benefited from indirect parental support.

Unlike many other raptors that rely strictly on flight for hunting, burrowing owls exhibit pronounced cursorial behavior. The pelvic limb morphology of *A. cunicularia* shows specific adaptations for terrestrial locomotion and burrowing, including elongation and slenderness of distal limb elements (Ilyinsky 2008). These adaptations allow the species to perform most of its vital activities on the ground. In the present case, the preservation of basic pelvic limb functionality, despite bilateral syndactyly (Fig. 1), together with these ecological adaptations, likely contributed to the maintenance of a satisfactory body condition score. However, the inability to fly, which led to the rescue and subsequent admission to the rehabilitation center, ultimately restricted the individual to the ground, increasing its vulnerability to predation and vehicle collisions.

In conclusion, this case of *Athene cunicularia* with complex congenital limb malformations supports a non-traumatic origin and highlights the relevance of wildlife monitoring in anthropogenic landscapes. Furthermore, it underscores the critical role of rehabilitation and triage centers in detecting sentinel abnormalities that may reflect underlying environmental degradation. As a recommendation for future research, complementary cellular-level analyses, such as the erythrocyte micronucleus test, could be applied in similar cases. Micronuclei represent chromosomal fragments or whole chromosomes excluded from the main nucleus during erythropoiesis, reflecting genotoxic damage not detectable by conventional karyotype analysis. This assay has been increasingly applied as a practical biomarker of genotoxicity in wild birds, with micronuclei frequency positively correlated with pesticide exposure in field studies (Baesse et al. 2015; Souto et al. 2025), and has demonstrated broad applicability across diverse wild bird species under field conditions (Quero et al. 2016). Incorporating this approach into future investigations at wildlife rehabilitation centers could strengthen the evidence linking congenital malformations in free-ranging birds to environmental xenobiotic exposure. 

## Data Availability

No datasets were generated or analysed during the current study.
